# The silver linings of Parkinson’s disease

**DOI:** 10.1038/s41531-022-00283-1

**Published:** 2022-03-03

**Authors:** Araceli Alonso-Canovas, Jos Voeten, Omotola Thomas, Larry Gifford, Jon A. Stamford, Bastiaan R. Bloem

**Affiliations:** 1 Movement Disorders Unit, Neurology Department, Hospital Universitario Ramon y Cajal, Madrid, Spain; 2 Radboud University Medical Center, Donders Institute for Brain, Cognition and Behaviour, Department of Neurology, Nijmegen, The Netherlands

**Keywords:** Parkinson's disease, Parkinson's disease

## Abstract

Parkinson’s disease (PD) is a neurodegenerative condition, characterized by motor, non-motor disability, and a reduced quality of life. Stimulated by a question raised by a person with PD, we posted an orienting survey on social media, asking whether there is possibly any “silver lining” (an upside) to having PD. Most respondents identified one or more positive changes, mainly a new focus in life, better coping skills, new activities, healthier lifestyle, and improved relationships with relatives and friends. This ability to perceive a silver lining of disease is in line with the concept of adversarial growth in illness, and positive health, which underscores resilience, self-management, and the ability to adapt. Importantly, not every respondent identified an upside to living with PD, so this is very much an example of personalized medicine. This is a delicate, difficult issue, and discussing the presence of silver linings may feel counterintuitive. However, exploring this issue may help people with PD and caregivers to better deal with the disease, and allow medical professionals to provide better support, to learn about coping strategies, to understand the degree of disease acceptance, and to enhance a healthier lifestyle. Further research should demonstrate whether addressing silver linings may impact positively on the outcome of PD and on the perceived quality of life. To facilitate this process, we have adapted a pre-existing silver lining questionnaire (SLQ-38) in light of the responses provided by people with PD, to offer a simple, feasible tool to further explore this issue in clinical and research settings.

I see ye visibly, and now believe

That he, the Supreme Good, to whom all things ill

Are but as slavish officers of vengeance,

Would send a glistering guardian, if need were

To keep my life and honour unassailed.

Was I deceived, or did a sable cloud

Turn forth her **silver lining on the night**?

I did not err; there does a sable cloud

Turn forth her silver lining on the night,

And casts a gleam over this tufted grove.

Comus: A Mask Presented at Ludlow Castle, by John Milton (1634)

## The concept of a “silver lining”

**Fig. 1:**
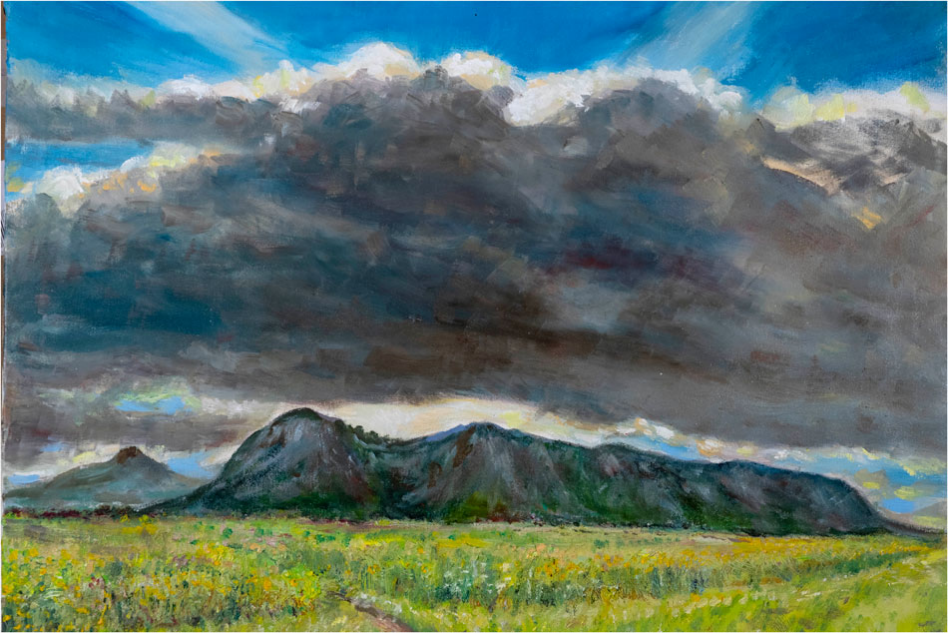
“A Silver Lining”, painted by Mr. Robin Broadhead, a retired paediatrician who lives with PD. “I painted this sitting on the verge of the Blantyre to Mulanje Road on the Tuesday 9th February. Mulanje Mountain is the highest mountain in Malawi and rises 10,000 feet from the Phalombe plain as the last gasp of the Great Rift Valley. I have for many years climbed and admired the mountain and seen it in its many moods. I have seen Mulanje after storms and when it is dramatically illuminated from behind by the break of dawn and has glowed warmly facing the setting sun in the West. The Mountain defiantly crouches below a dark and forbidding storm cloud and yet the sun behind the cloud remains blazing and the silver of light surrounds it as a silver lining. This is our rainy season and is a time of green growth and hope. Hope for a good harvest in the future weeks”.

The English phrase “There is a silver lining in every cloud”, derived from John Milton’s famous poem, means that a positive element may be found in every misfortune we experience (Fig. 1). This approach might be applied to Parkinson’s Disease (PD) and other chronic diseases causing disability: a positive attitude and appropriate management, aiming to enhance the preserved capabilities and to resiliently adapt and adjust to the new challenges, may help maintain and even increase the perceived quality of life^1–3^. However, usual healthcare is focused on the negative aspects of disability, and both clinicians and people affected with PD may be unaware that the attitude towards a disease and the adequate development or enhancement of coping strategies may play an essential role in prognosis and outcomes.

Recently, a person with PD (JV) asked his neurologist whether there was any upside to this diagnosis. The neurologist’s immediate reaction, being familiar with the tremendous impact that PD can have^4^, was one of denial. But on second thought, he realized that only persons living with PD themselves would be capable of answering this provocative question. To this end, we launched an orienting survey, with the main aim of identifying the first preliminary evidence for the presence of silver linings.

## Our orienting survey

We posted a video on social media (Instagram, Facebook, LinkedIn, Twitter) enquiring about the possible presence of a silver lining to PD (https://vimeo.com/497003406/699d88d101). We carefully phrased this question in a nuanced way, emphasizing it was merely an exploratory survey, and we clarified being open to a negative outcome. Explicit consent to use the answers was not requested from the participants, as the answers were collected from the public domain (social media) and were processed anonymously. The study protocol was submitted to the ethics committee “CMO Radboudumc”, registered as 2022-13456, and it was deemed exempt from further ethical approval. Responses were collected directly from replies to the video posts on the social media platforms.

We analysed all 138 answers that were received from January 20th to February 4th: of these, 113 (82%) included positive experiences, while 25 (18%) of respondents denied any. Most answers (85%) were provided by people with PD, except for answers submitted by attending neurologists (*n* = 9, 7%) or spouses/relatives (*n* = 8, 6%). This last subset of answers was comparable in sense and content to those provided directly by persons with PD, so we decided to include them in the analysis. This also helped to draw attention to the fact that silver linings may also be identified by both healthcare professionals and in particular also by direct family members, whose own quality of life can also be markedly affected. Answers were critically analysed, and the topics raised listed in non-predetermined categories, summarized in Table 1, along with examples (more details in Supplementary Table).

**Table 1:** Possible silver linings of Parkinson’s disease.

Silver lining (*n*, %)	Example
Better and improved focus in life, increased awareness (*n* = 73, 65%)	*I have learned the real value of life in all its beauty and complexity. The limitations of movement when Parky is at play, has given me an appreciation and joy of movement and the gratitude of modern medicine and the drugs and medicines, which have made my life possible and often pleasurable. I have learned patience and how to accept the loss of the illusion that I can control all situations. But the most important lesson I have learned is the grace of gratitude for life itself and all I have been given*.
New relationships, activities, interests (*n* = 46, 41%)	*I began to share my PD diagnosis with family, friends and to total strangers through interviews on radio and TV and by sharing my journey through my podcast, “When Life Gives You Parkinson’s.” I have impacted the world in a more positive way through my podcast and personal connections in three years with Parkinson’s than I have in 35 years in radio. I used to have a job, now I have a purpose*.
Engagement with the PD community (*n* = 20, 18%)	*I cycled through the Czech Republic and Slovakia (…), and through the Parkinson’s Association I meet other people again (…) I come into contact with numerous people I would never have seen otherwise, and in places I would never have been otherwise*.
Travelling (*n* = 14, 12%)	*When first diagnosed, I heard about ‘Parky Perks’* —*the unexpected good things that come out of having a diagnosis of Parkinson’s. For me, they are mainly in the form of the many wonderful people I have met and some fantastic experiences I have had (eg Cycling Vietnam to Cambodia and Land’s End to John O’Groats) that I would otherwise not have had*.
Improved relationship with family, friends (*n* = 39, 35%)	*Her husband told her that there are some diseases that you need to follow in love with, and he will just do it with her and for her*.
Better coping skills (*n* = 39, 35%)	*Even beyond Parkinson’s, this disease can teach one they’re able to handle much more than previously believed. This then can be extended to understand one has substantial control over their response to a situation even with limited control over its occurrence. Not a really advantage, but an opportunity to grow up*
Healthier lifestyle (*n* = 25, 22%)	*I started exercising, eating healthily and more consciously, meditating, and feeling better than I had in years*.
Improved self-esteem (*n* = 24, 21%)	*Developing a sense of self-compassion. More peace in myself, less fighting but letting it happen, I can handle it. I know my limits and how I can and want to deal with them*.
Improved professional life, starting new projects (*n* = 19, 17%)	*So I also found nice (voluntary) work again. And now I am also asked more and more to do other things. I feel very honoured with what I can do now. I have rediscovered a little world (just like the one I used to live in at P&G) in which I can do what I like and which is appreciated. I belong!*
Reduction of time devoted to work (*n* = 16, 14%)	*I am indeed more at home and enjoy my family and certainly my grandchildren more. That’s something I would never have done if I hadn’t had the disease, because you would have kept working 40* *h a week*.
Increased creativity, artistic skills (*n* = 7, 6%)	*I draw more freely and therefore more beautifully than before (…) I can sometimes make good use of the tremor when colouring larger areas*.

The left column depicts the main categories, while the right column provides an example for each category. The number of responses and proportion are shown between parentheses.

We were surprised by the many inspiring answers that we received. Examples included people who had found a better focus in their life following the diagnosis, or established new relationships, activities, or interests. Stimulating examples included the roles that some had assumed as advocate, to raise further awareness about the impact of PD, or to assist in fundraising. Others felt that the relationship with their spouse, family, or friends had become closer, or that their coping skills and self-esteem had improved. Indeed, PD is an exemplary condition where many affected people show a remarkable and creative ability to cope, by developing alternative motor programs to compensate for their deficits^5^. Some had engaged in a healthier lifestyle, including exercise or an improved diet^6,7^. Many also referred to a positive impact in their professional life: less time devoted to work so they could enjoy other meaningful activities, or starting new projects. A final silver lining related to a perception of increased creativity and heightened artistic skills, which had emerged following PD diagnosis. For at least some of these latter individuals, the effects of PD medication may be responsible, as a latent talent can be unveiled or enhanced by the dopaminergic stimulation^8^.

Not all answers were positive. Nineteen persons with PD (14%) answered that their life was worse after diagnosis, and six (4%) provided neutral answers (not better nor worse). Some were outright upset after reading the question about a possible silver lining of PD. These negative answers are in many ways understandable: these people indicated that they had never asked for PD, that the negative influence of this disease could be felt every day of their lives, and that all they wished for was a cure. We can see how these reasonable objections might have been influenced by several variables, such as disease severity, non-motor symptoms like depression, comorbidities, socioeconomic factors, the quality of family and healthcare support, or personality traits. These factors were not systematically ascertained in our orienting survey; this should be the topic of future work.

## Possible implications

We emphasize that this was merely an exploratory survey, not a research project with a predefined rigorous methodology. The sample size was small, and all sorts of bias could have influenced the answers. Depression, anxiety, and apathy are common in PD, and negative thoughts or emotions about the disease may be overwhelming. Such individuals likely never responded to our survey. Similarly, feelings of frustration, guilt, or anger may understandably have kept others from responding. We did not inquire about variables that possibly influenced the answers, such as age, gender, ethnicity, education, disease duration, comorbidities, medication, the quality and strength of family support or socio-economic status, and even spiritual beliefs. Many of these variables relate to the important topic of diversity in healthcare, which certainly deserves much more attention in future research^9^. Further work, therefore, needs to consider these elements related to diversity in relation to the possible presence of silver linings. Finally, this web-based survey, with an only limited time window for providing answers, only allowed us to reach a biased minority of the total PD population that is relatively more active on social media, while underserved populations due to issues such as poor education or poverty were likely underrepresented. Patient representatives who actively participate in PD associations, conferences, or research studies also typically represent a biased sample of highly motivated and presumably more educated persons living with PD. And we acknowledge that the prevalence of positive silver linings may well be higher among this selected sample of more active persons with PD. As such, the answers to this brief survey provide no ground truth, and the proportions of answers are unlikely to reflect the real-life population of people living with PD. Indeed, a study on the perspectives of the disease of by people with PD (43) and their caregivers (15), which included a question on the possible positive aspects of the disease, yielded only 5.5% positive responses^10^. Nevertheless, our intention was not to provide exact prevalence figures (or insight into the actual determinants), but rather to draw attention to the possible presence of silver linings in persons with PD. Metaphorically speaking, our orienting survey identified the clear presence of smoke, but further work remains needed to unravel the exact nature of the underlying fire.

Most respondents to our survey did perceive one or more silver linings to their PD diagnosis. This reminded us of the new concept of Positive Health, developed by Huber and colleagues^11^. These authors dismissed the 1948 WHO definition of health (“a state of complete physical, mental and social well-being”) as an idealistic, static, and perhaps even unrealistic concept^12^. Instead, they proposed an alternative definition of health as “the ability to adapt and to self-manage, in the face of social, physical and emotional challenges”. This concept enhances each individual’s potential to compensate for any limitations that diseases or other challenges may cause. In that regard, a person with PD would indisputably be defined as being sick according to the WHO definition, but could be seen as enjoying a Positive Health when this same person embraces one or more of the silver linings of PD. As such, it is challenging to question whether a person with PD who leads an active role as PD advocate is primarily someone living with a chronic disease, or first and foremost a person who has identified a new meaningful purpose in life? The concept of Positive Health also calls upon each individual’s own personal responsibility, thus supporting the idea of self-management. As such, being able to identify a silver lining as a useful coping skill has perhaps been neglected too much thus far. However, we should acknowledge that some concerns have also been raised, such as the substantial personal input that is required to be “healthy”, or the risk that people may seek medical attention too late^11^.

The concept of silver linings also relates to the field of positive psychology, which has extensively studied the “adversarial growth in illness”^13,14^. Chronic diseases may actually prompt positive changes in people’s lives. Importantly, the ability to perceive a silver lining of an illness has been linked to better mental health outcomes for several entities, such as heart disease and multiple sclerosis^13–15^. A scale called “The Silver Lining Questionnaire, SLQ-38” has been designed to explore this phenomenon^15^. SLQ-38 consists of five domains: improved relationships, increased appreciation of life, positive impact on others, increased inner strength, and changes in life philosophy. Indeed, the main silver linings identified by our unstructured study dealt mostly with these concepts. It would be interesting to see how SLQ 38 scores change during the course of PD, as a function of symptom severity and disease stage, or as symptomatic treatment is initiated or altered. Is an ability to identify silver linings more prominent in particular age groups, e.g. in the young? Would the silver lining scores mirror disease progression, or be restricted to a particular disease stage?

Interestingly, we should not automatically assume that people with an advanced disease are less likely to detect silver linings, as many of us have witnessed compelling stories of physically severely disabled persons with PD or with other diseases, who were able to keep a positive attitude towards life and even attained a high quality of life, a concept known as the “disability paradox”^1^. Specifically, the disability paradox refers to the sense of well-being and life satisfaction that may be perceived despite disabilities, meaning that quality of life is more than health-related quality of life, and depends, beyond physical challenges, on the social, psychological and spiritual being. In a semi-structured survey on 153 persons with disabilities, more than 50% rated their quality of life as good or excellent. A focus on preserved capabilities, the development of new coping strategies, and being an inspiration to others seemed to underlie these positive answers, while pain and fatigue were frequently reported by more negative respondents^1^. Further identifying these determinants represents another relevant question for future research.

Also in line with the positive health field, there is a body of literature exploring the possibility to flourish, or being able to improve some capabilities and functioning, after being diagnosed with a disabling condition, a concept called “ultrabilitation”: moving forward, around or beyond recovery of a particular function, seeking strength in the weakness^3^. For example, a recent paper identified falls in PD as a crucial clinical milestone, not from a negative point of view, but as a critical factor prompting a personal evolution in many possible ways. Specifically, the authors mentioned a readjustment of the perceived capabilities and safety measures, an inspiration to search for individual strategies or tips to avoid them, or to learn to fall safely, and to adopt a slower tempo in activities, thus allowing patients to cultivate a more serene and conscious presence^2^. In fact, many of the silver linings identified by our respondents are in line with these perhaps counterintuitive ideas (Table 1 and Supplementary Table). There are many examples of enhancement of preserved functions in the brain upon a static injury or following sensory loss, and such remarkable observations offer a biological plausibility for this framework^16^. Such adaptive neural plasticity likely also exists in the parkinsonian brain, where initially preserved brain areas can take over functions of the damaged nigrostriatal circuitry^5,17,18^. To grow better beyond a traumatic event is beautifully represented by the Japanese art of Kintsugi, which enhances the beauty of broken objects, repairing them with golden painting and other techniques^19^.

Finally, the degree of acceptance of the disease may be a key component of the ability to perceive a silver lining^14,20^. An appropriate acceptance of chronic illnesses is associated with less negative emotions, better physical and social functioning, and even adherence to treatments^21,22^. Several factors influence the acceptance of a chronic disease, but this issue has only been explored anecdotally in PD^22^. One study found that remaining professionally active and living in an intermediate population town were associated with a higher level of disease acceptance in PD^22^. Further research including socio-demographic and clinical variables is warranted to assess this complex issue.

We should also consider a perhaps somewhat counterintuitive explanation for the presence of silver linings, namely that these might actually reflect a form of disavowal. Having seen the edge of the silver linings, and specifically the constructive ways in which people dealt with their disease, we think this is an unlikely explanation for most respondents, but this issue does deserve further study.

We acknowledge that asking about a possible silver lining to a neurodegenerative condition is a delicate, difficult question. We mentioned how occasional respondents expressed very understandable objections against the very concept of silver linings, and these emotions must be considered very carefully when addressing this sensitive issue in daily practice. Also, even for those who responded positively, a silver lining is literally nothing more but a bright surrounding around an otherwise pitch-dark cloud, named PD. The PD field should always continue to search for strategies that can slow down or even arrest the progression of PD, to prevent the disease from happening in the first place, and to ultimately cure the disease for those who are already affected. But while we await for this to happen, we feel that the concept of silver linings–and the many incredibly encouraging stories we received–may offer new perspectives to further support the millions of people with PD worldwide, utilising an approach that has hitherto been largely unexplored.

We wanted to share our findings here, for several reasons: (i) as a tribute to those who had identified the silver linings in the first place; (ii) as a source of hope and inspiration for fellow persons with PD and their caregivers, who might find comfort in identifying their own silver linings as a way of better dealing with PD; and (iii) to help medical professionals in better supporting people with PD, by learning about their coping strategies, to understand the degree of disease acceptance, and to enhance a positive health approach, within a patient-centred precision medicine framework. In fact, when encountering a newly diagnosed person with PD, many clinicians already tend to emphasise several relatively more benign components of this diagnosis, aiming to offer hope and perspective; this includes a discussion of the relatively slow disease progression for most of the affected individuals, the typically long survival and the growing repertoire of treatment options. Clinicians can now consider to also address the issue of silver linings with such a newly diagnosed patient, obviously in a personalised approach tailored to the unique perspectives of each individual. Our approach may offer new arguments to build this positive focus which, importantly, coming comes from the experiences of real people with PD.

To support this approach and to facilitate further research, we here propose an abbreviated and more PD-specific version of the SLQ-38 (Table 2). This brief Silver Lining Questionnaire for Parkinson’s Disease (PD-SLQ) explores the five main domains of the original scale, but only through brief examples, and includes two newly added categories which were identified in our survey: ‘new relationships, activities and interests’; and ‘a healthier lifestyle’. This shorter, more open PD-SLQ may be immediately useful for clinicians to explore this issue in an orienting fashion with their patients in daily practice, but its psychometric properties should be tested properly in future research. Ultimately, we feel that it is important for every healthcare professional (regardless of the specific discipline) to be at least aware of the possible presence of silver linings, and that each professional can potentially address this issue, depending on the professional’s personal preferences and on the specific context of the consultation with each individual patient. However, looking at the versatile multidisciplinary team that is available to support persons with PD, we feel that mental health professionals may be positioned and equipped best to address this issue of silver linings in a careful and nuanced way^4^. We suspect that addressing this delicate but important issue of silver linings during medical consultations may help people with PD with accepting their disease. And if the concept is properly explored and engaged, it might improve the adherence to treatments, promote a healthier lifestyle, enhance autonomy and self-management, increase satisfaction and improve the quality of care and prognosis^13,21,22^. Moreover, it should be accounted for as a potentially modifying factor for the outcomes^22^. It has been proposed to consider happiness as a possible new outcome in PD trials^23^. Indeed, what matters most to our patients should be our priority too.

**Table 2:** Proposal for a brief Silver Lining Questionnaire for Parkinson’s Disease (PD-SLQ).

Thinking about your life after Parkinson’s disease (PD) diagnosis, please consider whether the changes listed in categories A to G have occurred at all in your experience. Please consider your agreement with each statement in a 1–5 scale:	(1) Strongly disagree (2) Disagree (3) Not sure (4) Agree (5) Strongly agree
A. Greater appreciation for life *PD gave me a new start, made me live life to its fullest, changed my focus in life. Now I appreciate and enjoy life more, I enjoy what I still can do. PD gave me an increased awareness. I learnt to accept the lack of control of situations, and uncertainty. I do not take health or any other thing for granted. Now I put things on perspective*	1 2 3 4 5
B. Changes in life philosophy *PD made me think about the true purpose of life. My religious/spiritual beliefs deepened because of PD*	1 2 3 4 5
C. Improved personal relationships *PD strengthened my relationships with others: spouse, family, and/or friends. PD made me more tolerant. PD made me less judgemental of others*.	1 2 3 4 5
D. Positive influence on others *I have been an inspiration to others. PD changed other people’s perception of me for the better. Other people appreciate me more because of PD*.	1 2 3 4 5
E. Personal inner strength *PD made me more aware of my strengths Improved my coping skills, made me more patient, empathetic, more humble, self-compassion, an opportunity to grow up. Improved resilience, self-esteem, learnt to put myself first. I am more humble, compassionate to others and myself. Learnt that I can cope with much more than I thought, more patience*	1 2 3 4 5
F. Acquisition of a healthier lifestyle *I made positive changes in my diet, increased physical exercise, I pay more attention to my habits and lifestyle*	1 2 3 4 5
G. Changes in personal relationships and activities *New activities and interests. Increased traveling. New professional projects. New relationships and engagement within the PD community. Involvement in PD advocacy. Reduction of time devoted to work. Increased creativity, more time devoted to creative activities*.	1 2 3 4 5
H. If there are other positive changes in your life you feel have not been mentioned, please outline these here:	

Our observations emphasize that the ability to see a positive perspective is very much a personalized issue, and not something that is present in every individual, let alone something that can be expected from everyone. In this present era of precision medicine, it is all the more important to recognize that PD is an incredibly heterogeneous disease, for which a “one size fits all” is inconceivable^4^. The concept of a silver lining is no exception to this. But if we do not acknowledge its presence and further explore this potentially important issue, we may be failing to provide the support and acknowledgement that many persons with PD need and deserve. Our recommendation for healthcare professionals, first, to be aware of the possibility of silver linings of living with this disease. Second, to motivate clinicians to judiciously address this issue with persons with PD in their daily practice, perhaps using the brief PD-SLQ scale to guide the discussion. Finally, to stimulate further research on this complex issue, which may help us to better understand some of the heterogeneity of the experiences of people with PD.

### Reporting summary

Further information on research design is available in the Nature Research Reporting Summary linked to this article.

## Supplementary information

Supplementary material

REPORTING SUMMARY
