# Supplementary material for: The silver linings of Parkinson’s disease
Source: NPJ Parkinsons Dis. 2022 Mar 3;8:21. doi: 10.1038/s41531-022-00283-1 (PMC8894354; doi:10.1038/s41531-022-00283-1)
Supplement: Supplementary file 1 — Supplementary material [file 41531_2022_283_MOESM1_ESM.pdf]

SUPPLEMENTARY MATERIAL

Further selected examples of some possible silver linings of Parkinson's disease, as reported by patients (mostly), their attending neurologists, spouses or relatives, classified considering the seven proposed domains of SLQ-PD.

A. Greater appreciation for life

Well although I cannot be grateful to "Parky" as I call him directly for anything, I am appreciative of what He has indirectly taught me. I have learned the real value of life in all its beauty and complexity. The limitations of movement when Parky is at play, has given me an appreciation and joy of movement and the gratitude of modern medicine and the drugs and medicines, which have made my life possible and often pleasurable. I have learned patience and how to accept the loss of the illusion that I can control all situations. But the most important lesson I have learned is the grace of gratitude for life itself and all I have been given.

I learned a positive mind-set makes the difference. Practicing a lot of sports and having fun helps. I can sincerely say I am happier than I was before diagnosis. My advice: enjoy now and accept the uncertainty of the future.

PD is a reflection of life. Life, even in the best possible state of health, is a degenerative process with no solution. PD helps bear this in mind, and put things into context. The fact that PD cannot be hidden, the need to talk about it, makes you more humble, better accept human condition, and more solidarity with people around you.

Even beyond Parkinson's, this disease can teach one they're able to handle much more than previously believed. This then can be extended to understand one has substantial Control over their response to a situation even with limited control over its occurrence. I can drive from internal desires rather than being driven by external forces. Understanding this has been the most valuable lesson of my life.

The realisation that good health is not something we can take for granted has made us more aware of the "ordinary" things and enjoy them more.

It took me a while to work out what I needed to do and change in order to keep myself as healthy as possible. I have mapped out what gives me dopamine (in addition to a minimal dose of sinemet) and what costs me dopamine. So now I live very consciously and I use my energy sparingly.

Since the diagnosis of Parkinson's, but especially after taking a driving test at the CBR, I realise that riding a motorbike will end one day. So it is actually quite special to be able to drive a motorbike, even if you have Parkinson's disease. As long as it is still possible and responsible, I enjoy every motorbike ride twice as much because I know that one day, I will no longer be able to do it. (…) I experience things much more consciously, that I no longer rush through things and that I don't take everything for granted. I live in the now and enjoy it!

Having time is prominent from now on. P has given me a crash course in time management. The things I didn't have time for before or didn't want to have time for, turn into moments that are worthwhile. A listening ear, a moment of reflection or rest, a conversation.

A. Changes in life philosophy

No, it doesn't make life more fun or easy. It doesn't compensate either but that awareness, awareness and conscious experience is so beautiful and precious…

More intense religious feelings: a greater connection with God

It forces the PD patient and their families to slow down and live life more consciously - prioritizing in what matters most.

The benefit of the positive health approach for me is not to look at myself as a victim but to look at the possibilities I still have. 'Always look on the bright side of live', knowing that there is a dark side. It is actually more about mental well-being and the healthy balance between body, mind and emotion.

B. Improved personal relationships

Her husband told her that there are some diseases that you need to follow in love with, and he will just do it with her and for her.

The diagnosis and its consequences have brought my wife and me much closer together; "we are going to face this challenge together".

My three kids understand the situation well, and I feel proud of the dignity they show in living with the disease. My 8 year old daughter explains to her friends her daddy's handshakes, but he is alright, in such a tender way...

You also have a different bond with your children, you show more interest in each other, not that this wouldn't be the case otherwise, but it is extra now.

We live now more consciously, quietly and with much more attention to each other.

C. Positive influence on others

The thing that drives me on is the need to make a difference. To know that you have, in however small away, changed people's lives. It might just be to make some think differently. It might be practical help. It might affect one person or it might affect 1 million. It doesn't matter. It's all about making ripples on the water surface. Because ripples go on forever.

I began to share my PD diagnosis with family, friends and to total strangers through interviews on radio and TV and by sharing my journey through my podcast, "When Life Gives You Parkinson's." I have impacted the world in a more positive way through my podcast and personal connections in three years with Parkinson's than I have in 35 years in radio. I used to have a job, now I have a purpose.

I was driven to do something good for Parkinson's. And it turned out to be something that I liked. And as it turned out, it seemed like something that was on my path. I can put a lot into this. And use the marketing again, which is what I like so much. So I also found nice (voluntary) work again. And now I am also asked more and more to do other things. I feel very honoured with what I can do now. I have rediscovered a little world (just like the one I used to live in at P&G) in which I can do what I like and which is appreciated. I belong!

I became involved in various ways for Parkinson's and that has brought me a lot. Many new contacts, much appreciation, new friends, a very personal identity. I used to be a nurse (...) Now I am a pioneer, a source of information and a role model in my environment.

D. Personal inner strength

Parkinson's makes you feel out-of-control of your own body. But, when I realized the one thing I can control is how I react to the Parkinson's – a new world opened up for me.

To freeze or not to freeze, it is just a state of mind: relax and let it go.

Even beyond Parkinson's, this disease can teach one they're able to handle much more than previously believed. This then can be extended to understand one has substantial control over their response to a situation even with limited control over its occurrence.

PD changed the way I think: now I put things on perspective, I think I am more patient, more open minded, less of a whiner

PD taught me I cannot give myself a break. Physically and mentally, I cannot stop. If I stop challenging myself, making progress, reaching beyond my limits, I will lose.

Parkinson's showed me how mental strength, determination and stubbornness can help you achieve new goals! And I still do!!!

I try to enjoy more the things I can do, and yes there are also things I can't do any more or less well and I accept that.

Not that I have become the ideal partner now, I just notice that there is a positive evolution in my personality.

After a PD diagnosis it makes you re-evaluate life. The positive side is that I have more empathy for others. Everyone has something going on in their lives. Be kind.

Not a really advantage, but an opportunity to grow up.
I feel proud of myself when resilience overcomes physical symptoms and weariness.

Developing a sense of self-compassion. More peace in myself, less fighting but letting it happen, I can handle it. I know my limits and how I can and want to deal with them.

It was a slap in the face but caused a huge turnaround. I finally started to embrace myself, and to put myself first.

I had to recalibrate my self-esteem. I was actually quite insecure in the beginning (...) I derived my right to exist from being a good mother, daughter, partner, friend, employee and colleague (...) To my great emotion, I receive confirmation from many quarters that I am actually appreciated for my personality and being there (...) I am aware of my own value and what it means to others.

Better fitness, strength, confidence & more adventurous.

I feel all day that I have Parkinson's, but I am glad that despite the illness I also feel very good. Apparently I can do something about it myself, and that makes me proud.

E. Acquisition of a healthier lifestyle

Two years after my diagnosis (47 years old) I started to exercise fanatically. When I started, walking 1.5 km was the maximum I could do. Two years later I completed the 4 days of Nijmegen.

I started exercising, eating healthily and more consciously, meditating and feeling better than I had in years.

I always lived healthy but now even healthier by leaving out sugars and following some kind of Parkinson's diet. Which makes me feel very good. I walk and sport every day because that also made fitter. I also enjoy nature much more because of it.

F. Changes in personal relationships and activities

In the UK, we also have the term 'Parky Perks'. When first diagnosed, I heard about 'Parky Perks' - the unexpected good things that come out of having a diagnosis of Parkinson's. For me, they are mainly in the form of the many wonderful people I have met and some fantastic experiences I have had (eg. Cycling Vietnam to Cambodia and Land's End to John O'Groats) that I would otherwise not have had.

New inspirational friends. Extra initiative. New purpose. Helping others. More compassionate.

We wanted to stop working early and start travelling. Immediately after the diagnosis, 10 years ago, we went out regularly. We let ourselves be guided by what was still possible, so the most exhausting trips first. Tibet at high altitude, far into the Amazon region by canoe, visiting remote and difficult to access camps in Africa by four-wheel drive, where the animals roamed around the tent.

The amazing people, the traveling (I would never have driven around Oregon in a red Mustang convertible with my mum without PD), the shift in perspective on your priorities

I cycled through the Czech Republic and Slovakia (...), and through the Parkinson's Association I meet other people again (...) I come into contact with numerous people I would never have seen otherwise, and in places I would never have been otherwise.

I am happier, feel more alive, and I dare to start new projects (...) such as composing music, a theatre course, and I have plans and ideas to start writing. Probably these projects remain as simple hobbies, but I am enjoying it so much…

I am indeed more at home and enjoy my family and certainly my grandchildren more. That's something I would never have done if I hadn't had the disease, because you would have kept working 40 hours a week.

Less stress. Early retirement.

Since my partial disablement, I still work half days. Partly thanks to my employer's willingness to offer modified work and my motivation to continue working, I no longer have to give up full days and can go home after half a day at work. This allows me to keep on working, something I enjoy doing, for longer.

I'm sure I would have continued to undertake business until my 67th year (with all the stress that comes with that) if I hadn't had Parkinson's disease

I discovered I am much more creative than I thought: I paint and write a lot now.

I draw more freely and therefore more beautifully than before (...) I can sometimes make good use of the tremor when colouring larger areas.

My practice and composing music with my guitar became extremely creative and prolific

G. If there are other positive changes in your life you feel have not been mentioned, please let us know:

Explanation of physical symptoms

(upon diagnosis) The pieces of the puzzle all fall into place. Various symptoms that had been developing over time suddenly appear to be well explained and can be categorised as Parkinson's. They have not become less severe, but they have become more noticeable.
They have not become less but it gives me the peace of mind that they can be seen from that perspective. And also that they can be addressed and/or easily accepted.

The diagnosis brought shock, sadness and anger, but also clarity, medication and physiotherapy. And in the end, it made everything much more bearable.

Making the right diagnosis and giving it a name seems like the basis for moving forward, seeking help, accepting things, knowing you're not the only one. Looking for information about your illness, finding the right first responders.

Family decisions

We forced my husband's Parkinson's disease to look at the future. Decided we don't do that pessimistically. We chose foster children. Are home more often, husband is in bed early so aren't often out the door (more) and these factors contributed to the choice. A lot of people have said would you, you don't know what the future looks like. No, that's right, but nobody knows that! We have had two foster daughters for a year and a half now, I'm home. Of course it's spicy sometimes but definitely an enrichment. Of life and of yourself!

When my dad got the diagnosis of Parkinson's (…) we were able to build a new house next to the farm (...) It was designed on the future (climate neutral and wheelchair friendly) so that my dad could stay home as long as possible. In the beginning my father helped us a lot on the property and later the roles were reversed when things went backwards with him. Then we started helping him more with the daily things. Thanks to my mother's enormous effort and love, he was able to live in the place he loved as long as possible. Next Monday it will be 4 years since my father passed away and we are still looking back on a beautiful period that we all kept things running. And still now we live on this fantastic farm and my mom in the house on the left of the picture. So like you said in the call: you don't give anyone this horrible disease but it has also created a great living situation that we still enjoy so much to this day.

Miscellanea

A higher climatic conscience, and focus on sustainability, thinking of how pesticides and pollution have an influence on de development of Parkinson’s disease

Awareness and concern for a heathier environment,

Increased interest in neuroscience, and how our brains work, and in PD research

Benefits in taxes, parking and other financial upsides
